# Why Is Hygiene Not Taught in Medical Colleges?

**Published:** 1878-01

**Authors:** 


### WHY IS HYGIENE NOT TAUGHT IN MEDICAL COLLEGES?

We hear and talk much of the progress of sanitary science, and there is no doubt but that hygiene is securing much more attention on every hand than in former times.

The advances, however, of this science are not commensurate with the importance of the subject, nor with the progress of enlightenment that exists in regard to the cause of various diseases. In fact, those who should urge onward this department are too frequently the least informed on the subject.

Physicians, as a rule, are not informed to any great degree concerning personal and public sanitation. But is it strange? We have before us several "annual announcements" of medical colleges, but in very few of them is hygiene taught either during the preliminary or the regular course. In the University of Pennsylvania a Chair of Hygiene exists in the auxiliary course, but, unhappily, only a small minority of the regular medical class attend it. During the medical student's college course how much time is given to hygiene? How much of the clinical hour? How much of the lectures on the practice and theory of medicine? What is said upon the subject is incidental—may we not say *accidental?* It is probable that the professor of practice of medicine refers to good hygiene in the opening remarks of the treatment of a disease; but hygiene is a subject of such magnitude that the qualifying word "good" leaves a very dreamy sort of an idea of how to bring into use the powers of nature.

Is it any wonder that a certain popular lectures exclaims, "If one were to start out in pursuit of information upon the laws of health, and he should consult thirty-six persons, twelve doctors, twelve clergymen, and twelve intelligent grandmothers, the doctors would give him the least knowledge?" No doubt we can select the proper pill to swallow, but, as the same gentleman says, "If you wish to know how to avoid being sick, or you are well and wish to know how to keep so, the first intelligent person of mature years you may happen to meet will give you more practical wisdom than your own family physician." There is a substratum of truth in his further statement that in looking on the sick side of a man the doctors are very apt to know less about the well side of him than other folks.

This may be an exaggerated picture, but yet, in the main, is it not only too true? How often in our medical journals do we see original communications, correspondence and notes on health? Is not sanitary science considered a specialty? The work of a certain few? It should not be so, and yet it is hard to conceive how the disgrace can be obviated uuless hygiene becomes a regular "chair," or, at least, it is taught during the summer and preliminary courses. It is just as interesting and important as a course upon microscopy, symptomatology, laryngoscopy, etc. These are dignified with a lectureship. Why not hygiene?

*Medical and Surgical Journal.*

